# Bibliography

**Published:** 1916-05

**Authors:** 


### BIBLIOGRAPHY

NOTES ON DENTAL METALLURGY. This is a very interesting work on the application of metallurgy to dentistry, especially intended for dental students. The book is written by W. Bruce Hepburn, L.D.S., of Glasgow, Scotland, and this, the second edition, is printed in this country by Wm. Wood & Co., of New York. The new edition is up to date in its treatment of amalgam alloys, and it contains a number of new alloys that promise to be useful adjuncts to practical dental problems. This book is valuable because of the style in which it is written. There is brevity and simplicity of statement, and yet each subject seems to have been carefully studied and recorded in succinct but intelligible language. It makes very interesting reading. We know of no work on this subject that will give one a clearer review of metallurgy applied to the dentist's needs and which is so attractively written. It is a book for students and practitioners who want condensed and reliable information. One feels that on some subjects he would like more extended treatment, but this, of course, should not be expected in a book of this character. It is an excellent book for students and practitioners.

DENTAL STATE BOARD QUESTIONS AND ANSWERS, by R. Max. Goepp, M.D. This is the second edition of Dr. Goepp's book on State Board Questions, and while no extensive additions seem to have been made, the author has brought the book more nearly up to date on such subjects as have been more actively under review during the last two or three years since the former revision. Such books are subjects of suspicion to a more or less extent, as they are likely to be used by students in college as "ponies" to cram for examinations. The general intention is that they will serve to acquaint men with the kind of questions that will be asked by various state board examiners, so that they can more readily prepare for such examination.

This is, of course, a legitimate reason for such a book, as many practitioners change their residences from one state to another, and after being out of college for several years they naturally do not retain the more technical knowledge of the scientific subjects. There should, however, be little use for such books on the more clinical subjects, and it would seem that here more stress should be placed by examiners, especially for experienced practitioners. This book is the best of its class, and is published in the usual good style of the W. B. Saunders Co., of Philadelphia.

APPLETON'S MEDICAL DICTIONARY. This is a new candidate for favor in the dictionary field. It comes from the pen of Dr. S. E. Jelliffe, A.M., M.D., Ph.D., of the New York Post-Graduate Hospital and others; and is published by the house of D. Appleton & Co., N. Y. The book is not so large and bulky in form as the older works because it has dropped all obsolete and historical words; and it seems to be an effort to incorporate only words of stable and newer usage. We notice that it gives much valuable space like its predecessors to names of proprietary remedies, which it can not properly define for lack of knowledge as to their composition. We note one dental remedy which is defined—dentola—which we never heard of before and which is certainly not a recognized remedy in dental therapeutics. There are a large number of medical words of a similar kind which it does not seem necessary to include in a working dictionary for medical and dental students. Most of the dental terms included are fairly well defined, but like other medical dictionaries dental terms have not been given adequate consideration. We suppose the reason is that no dictionary made for medical purposes would want to be encumbered with dental technical terms. A useful addition in the form of an appendix contains much valuable information in the way of tables, formulae, weights and measures. It is a very handy and well made book.

